# Reproducibility of Her2/neu scoring in gastric cancer and assessment of the 10% cut-off rule

**DOI:** 10.1002/cam4.365

**Published:** 2014-12-16

**Authors:** Hans-Michael Behrens, Viktoria S Warneke, Christine Böger, Nele Garbrecht, Eva Jüttner, Wolfram Klapper, Micaela Mathiak, Ilske Oschlies, Ursula Rudolph, Christiane Stuhlmann-Laeisz, David Trick, Christoph Röcken, Peter Hufnagl

**Affiliations:** 1 Department of Pathology, Christian-Albrechts-UniversityKiel, Germany; 2 Department of Pathology, Charité University HospitalBerlin, Germany

**Keywords:** Cut-off value, gastric cancer, Her2/neu, virtual microscopy, visual perception

## Abstract

The application of Trastuzumab on gastric cancer patients is based on Her2/neu immunostaining. The testing method relies on visual estimation of both membranous staining intensity, and positive tumor ratio with respect to a 10% cutoff. We evaluated the effect of inter- and intraobserver variations of both factors on therapeutic decision, especially if the positive tumor ratio hovers around the 10% cutoff. Ten pathologists scored 12 Her2/neu immunohistologically stained whole sections of gastric cancer. Applying the common rules for Her2/neu testing for gastric cancer, they separately noted the strongest identifiable staining intensity and the corresponding positive tumor ratio. Scoring was done repeatedly using the microscope, plain virtual microscopy, and virtual microscopy with a manual outline drawing function. Agreements on the strongest identified staining intensities were moderate. Overall concordance correlation coefficients of positive tumor ratios ranged from 0.55 to 0.81. Reproducibility was not improved by virtual microscopy. Pathologists have a good ability to estimate ratios of clearly demarcated areas, but gradients in staining intensities hinder reproducible visual demarcation of positive tumor areas. When hovering around the 10% positive tumor ratio cutoff there is a risk of misinterpretation of the staining results. This could lead to a denial of Trastuzumab therapy. Assessment of Her2/neu expression should be carried out by experienced pathologists because they can more reproducibly rate membranous staining intensities. The low reproducibility of positive tumor ratio is inherent in the testing method and cannot be improved by virtual microscopy. Therefore, we propose to reconsider the 10% cut-off limit.

## Introduction

Gastric cancer (GC) is the second most common cause of cancer-related deaths in the world. Approximately 70% of the patients have already lymph node metastases at the time of the diagnosis. Complete resection of the primary tumor with D2-lymphadenectomy offers the only chance of cure in the early stage of the disease. Survival of more locally advanced GCs was significantly improved by the introduction of perioperative, adjuvant, and palliative chemotherapy.

Recently, Her2/neu was introduced as a predictive biomarker for the treatment of GC with trastuzumab. Trastuzumab is an antibody targeting Her2/neu and is applied in combination with chemotherapy for the treatment of Her2/neu positive advanced GC 1. The Her2/neu status is assessed by surgical pathologists using tumor tissue obtained by biopsy or by resection and immunohistochemistry in combination with in situ hybridization. A GC is Her2/neu positive, when ≥10% of the tumor cells show strong circumferential, lateral, or baso-lateral immunostaining, or when ≥10% of the tumor cells show weak to moderate circumferential, lateral, or baso-lateral immunostaining in combination with HER2/neu gene amplification.

An almost overwhelming number of studies demonstrated the robustness of the Her2/neu testing (for a review see also 2). However, the assessment of Her2/neu status is hampered by (1) its heterogeneous expression in GC, carrying the risk of a sampling error 3–14, and (2) by the surgical pathologist's visual perception of what is below and above 10%. In a previous study 14, we evaluated the risk of sampling errors in specimens of biopsy size, which may be caused by heterogeneous overexpression of Her2/neu in GC. Tissue microarrays served as “biopsy procedure” and were compared with 454 whole tissue sections obtained from the same paraffin blocks used for the generation of tissue microarrays. The Her2/neu status was determined according to GC scoring system 15 by two independent observers using immunohistochemistry and in situ hybridization. In that study, we identified the particular problem of visual assessment of positive (≥10% positive tumor cells) or negative (<10%) when the amount of positive tumor cells is near the cut-off value of 10%. This motivated us to design an experiment to further validate the problem of the cut-off value and assess the agreement of Her2/neu scoring between multiple observers and trying to find a method leading to more reproducible results.

Our experiment now assesses the agreement of the strongest identifiable staining intensity as well as the positive tumor ratio between pathologists and methods, using (1) the standard microscopic method, (2) virtual microscopy, and (3) virtual microscopy with additional assistance for outlining tissue areas.

## Materials and Methods

### Participants

Ten pathologists were recruited as participants. Six had been practicing as board-certified pathologists for 4–10 years (median 5.5 years), and four as residents for 3–6 years (median 4.5 years).

### Samples

Twelve Her2/neu immunohistochemically stained large sections (monoclonal antibody 4B5; Roche Diagnostics GmbH, Mannheim, Germany) were selected from a previous study of 454 cases of GC 14. Since we wanted to assess reproducibility of positive tumor with respect to the 10% cut-off threshold, we selected cases from the previous study that had been problematic in whether the positive tumor ratio was above or below 10%, and added some cases with a positive tumor ratio in higher ranges. Additionally, for all 12 cases Her2/neu gene amplification was evaluated using the HER2-SISH double-labeling in situ hybridization system and the Ventana BenchMark XT automated slide staining system (all Roche Diagnostics GmbH). Identities or further data of the 12 cases were not known to the pathologists. Characteristics of the cases are given in Table S1.

### Virtual microscopy

The samples were scanned using a Leica SCN400 microscopic whole-slide scanner (Leica Biosystems, Nussloch, Germany) at its maximum, nominally 40 times magnification. In the scanned images, pixel-to-pixel distance represents 0.26 *μ*m. Images were exported from the scanner system into files of Leica SCN format. For performing the computer-assisted parts of the experiment, a viewer program was written to display images of Leica SCN file format. This gave us the flexibility to create the screen layout, user interaction, assistance tool, and calculation routines we needed for our experiment. A view of the program is depicted in Figure S1.

### Procedure

The 10 pathologists rated each of the 12 slides repeatedly using three methods (summing up to 36 ratings per pathologist and a total of 360 ratings in the entire study). The pathologists were asked for two values: First, the raw value of the strongest immunostaining they could identify within the specimen on the scale 0–3 analogous to the rules described by Rüschoff et al. 15 (0, no reactivity; 1, barely visible; 2, weak to moderate; 3, strong), but still without applying the cut-off rule of 10% positive tumor ratio; Second, an estimation of the ratio of only this strongest stained tumor tissue to total tumor tissue (0–100%). The intent was to spot the cases where the 10% cut-off rule would have had to be applied. In order to minimize memorization at least 2 months were allowed to pass between rounds.

### Method 1: Microscope

First the pathologists performed a Her2/neu scoring of the samples using their own, familiar microscope as in daily work. They sequentially received the set of 12 slides along with a questionnaire asking for the strongest staining they could identify, applying also Rüschoff's magnification rule 15, as well as the positive tumor ratio.

### Method 2: Virtual microscopy

Rating of staining intensity and positive tumor ratio was repeated on the scanned slides, utilizing our viewer program. The program was configured to offer only viewing functions (zoom and move), but no additional aid. Magnification buttons (40×, 20×, 10×, 5×) allowed to switch magnification in order to imitate the handling of a conventional microscope.

### Method 3: Virtual microscopy with area outlining assistance

In the third round of rating we first repeated the estimation of staining intensities with the viewer program. For measuring positive tumor ratio we tested an alternative, computer-assisted method. We extended the viewer program by a polygon line drawing function. This was used by the pathologists to separately trace the outlines of total tumor tissue and positive tumor tissue (Fig. S1A). Finally they were presented a homogeneously color-filled sketch of the outlines they had drawn (Fig. S1B), and were asked to visually estimate the positive tumor ratio from this sketch. The drawing was saved in a file, so we could calculate the exact ratio afterward for comparison.

### Statistics

Statistical analyses and tests were conducted using SPSS version 20 (IBM Corporation, Armonk, NY) and R version 3.0.1 (R Foundation for Statistical Computing, Vienna, Austria). Intrarater agreements of categorical variables (immunoreactive scores [IRSs]) between two rounds, respectively, were determined using the kappa test. A kappa value of 0.01–0.20 was considered to be slight agreement, of 0.21–0.40 to be fair, of 0.41–0.60 to be moderate, of 0.61–0.80 to be substantial agreement, and of 0.81–1.00 to be almost perfect agreement 16. Interrater agreements of categorical variables (IRSs within one round) were calculated using Fleiss’ kappa test 17, which is appropriate for multiple observers rating multiple subjects, using the irr package for R 18. Agreements between continuous variables (positive tumor ratios) were calculated using the overall concordance correlation coefficient (OCCC) 19 which is implemented in the epiR package for R 20.

### External quality assurance

Both HER2/neu testing methods, immunohistochemistry as well as in situ hybridization, were certified successfully in 2013 by the quality assurance program of the German Society of Pathology and the *Bundesverband Deutscher Pathologen e.V*.

## Results

**Figure 1:**
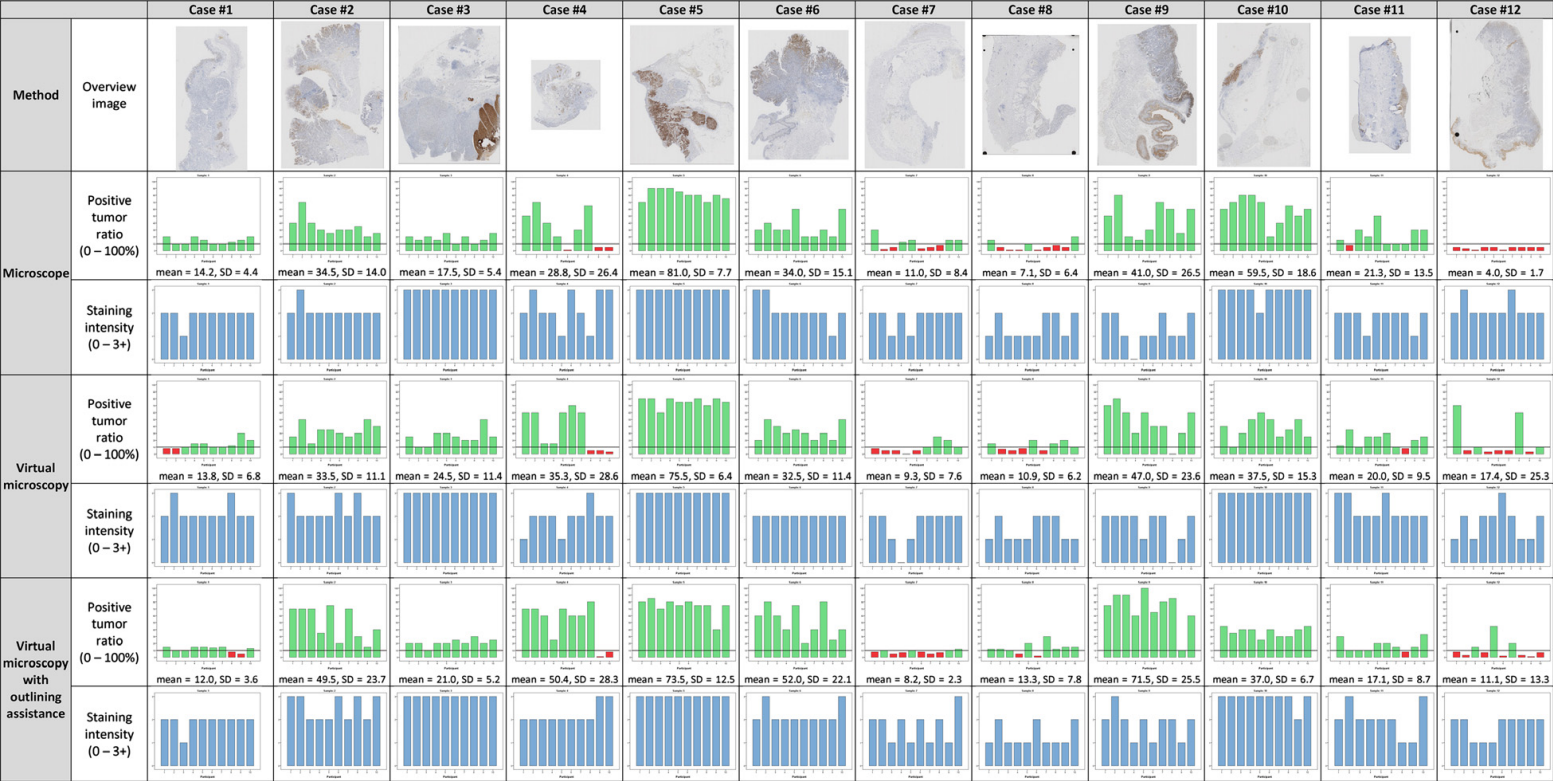
Overview of all ratings, grouped by the rating method (microscopy, virtual microscopy, and assisted virtual microscopy.) Each diagram shows 10 bars, corresponding to the ratings of one specimen by 10 pathologists, using the indicated method. Ratings are shown separately for positive tumor ratio (green bars ≥10%, and red bars <10%), and staining intensity (blue bars). Below the diagrams, mean and standard deviation of positive tumor ratio of the 10 ratings are indicated. The top row shows overview images of the 12 scanned slides.

The pathologists’ individual ratings of staining intensity (0, 1+, 2+, or 3+) and positive tumor ratio (i.e., percentage of stained tumor tissue, 0–100%) were plotted as bar diagrams to illustrate the interobserver variations (Fig.1).

We quantified interobserver agreements separately for each method using statistical tests appropriate for the situation of multiple observers (pathologists) rating multiple subjects (slides). For staining intensities (ordinal scale) we used Fleiss’ Kappa test 17, which takes a value between 0 (no agreement) and 1 (perfect agreement). For positive tumor ratios (continuous scale) we used the OCCC 19, which also takes a value between 0 and 1. Additionally, we calculated the standard deviations of positive tumor ratios per slide (Fig.1) and averaged over all slides. Table1 summarizes the interobserver agreements.

**Table 1:** Interobserver agreements between pathologists using one method of rating

		Agreement of positive tumor ratio			Agreement of staining intensity
Method		OCCC	Mean of standard deviations [%] (range)	Number of specimens with 10% cut-off disagreements	Fleiss’ kappa
1. Microscope		0.682	12.1 (1.7–26.5)	4	0.44 (*P* < 0.001)
2. Virtual microscopy		0.551	13.7 (6.2–25.3)	7	0.45 (*P* < 0.001)
3. Virtual microscopy with area outlining assistance	Visual estimation by the pathologists	0.672	13.1 (2.3–28.3)	6	0.40 (*P* < 0.001)
	By computation of outlined areas	0.694	12.3 (1.9–25.2)	7	

Positive tumor ratios were compared using the mean of standard deviations, overall concordance correlation coefficient (OCCC), and number of discordant ratings with respect to the 10% cutoff. Staining intensities were compared using Fleiss’ kappa test.

We took the consensus Her2/neu status that was rated by a panel of three pathologists from the previous study 14 as reference with which we compared each of the ratings in this study. This allowed identification of false-negative ratings due to the following causes: (1) Staining intensity was rated as strong (3+), but underestimation of stained tumor ratio (<10%) led to negative Her2/neu status. (2) Staining intensity was rated as moderate (2+) and stained tumor ratio was underestimated (<10%), thus no SISH would have been carried out. (3) Staining intensity was underestimated as 1+ or 0, but previously was 3+ or 2+ in combination with a positive SISH result.

### Results from Method 1 (Microscope)

When using the microscope, we found a 12.1% mean standard deviation of positive tumor ratio, ranging from 1.7% to 26.5% over the 12 cases, and an OCCC of 0.682. Four cases were discordantly rated with respect to the 10% cutoff. Interobserver agreement of the rating of staining intensity showed a moderate agreement (Fleiss’ kappa was 0.44). A comparison of the IHC findings with the reference results illustrates the risk of underestimation of Her2/neu immunopositive tumor area (<10%): six ratings underestimated the tumor area (<10%) and no SISH would have been carried out leading to denial of medication, although HER2/neu gene amplification was found by SISH. Thus, Her2/neu 2+ cases are extremely sensitive to misrating at the 10% cut-off point (Table2). Interestingly, underestimation of staining intensity even led to 10 false-negative ratings in five cases. Combination of both causes showed 17 false-negative ratings in five cases. In relation to the 90 ratings that were performed on the nine SISH-positive cases, this corresponds to a false-negative rate of 19%.

**Table 2:** Numbers of false-negative ratings and cases due to underestimation of positive tumor area and/or staining intensity

	Number of false-negative ratings/number of cases affected		
	Microscope	Virtual microscopy	Assisted virtual microscopy
False-negative because positive tumor ratio was underestimated (<10%), while staining intensity was 2+ or 3+ and SISH was positive	6 (7%)/2	6 (7%)/3	6 (7%)/2
False-negative because staining intensity was underestimated (0 or 1+), while positive tumor ratio ≥10% and SISH positive	10 (11%)/5	2 (2%)/1	7 (8%)/4
Total false-negative rate because positive tumor ratio or staining intensity or both were underestimated, while SISH was positive	17 (19%)/5	12 (13%)/4	16 (18%)/4

Numbers in parentheses denote the false-negative percentage related to the 90 ratings per method that were made for the nine cases with positive reference Her2/neu status (10 pathologists times nine positive cases). Interestingly, all these cases showed HER2/neu gene amplification, illustrating the risk of underestimating the percentage of Her2/neu-immunostained tumor area or intensity of immunostaining.

### Results from Method 2 (Virtual microscopy)

Applying plain virtual microscopy did not improve the reproducibility of the positive tumor ratio. We found 13.7% mean standard deviation of positive tumor ratio (range 6.2–25.3%), and an OCCC of 0.551. Seven cases were discordantly rated concerning the 10% cutoff. Staining intensities showed a moderate agreement (Fleiss’ kappa was 0.45). Comparison with the reference consensus Her2/neu status revealed six false-negative ratings in three cases due to underestimation of positive tumor ratio, two false-negative ratings in one case due to underestimation of staining intensity, and combined 12 false-negative ratings in four cases (Table2).

We noticed that cases having low standard deviations of positive tumor ratio tend to express a black-and-white staining pattern with sharply demarcated borders (e.g., cases 1, 3, and 5). On the other hand, cases with a more gray scale or heterogeneous expression pattern show a high standard deviation (e.g., cases 2, 4, 6, and 9).

### Results from Method 3 (Virtual microscopy with area outlining assistance)

**Figure 2:**
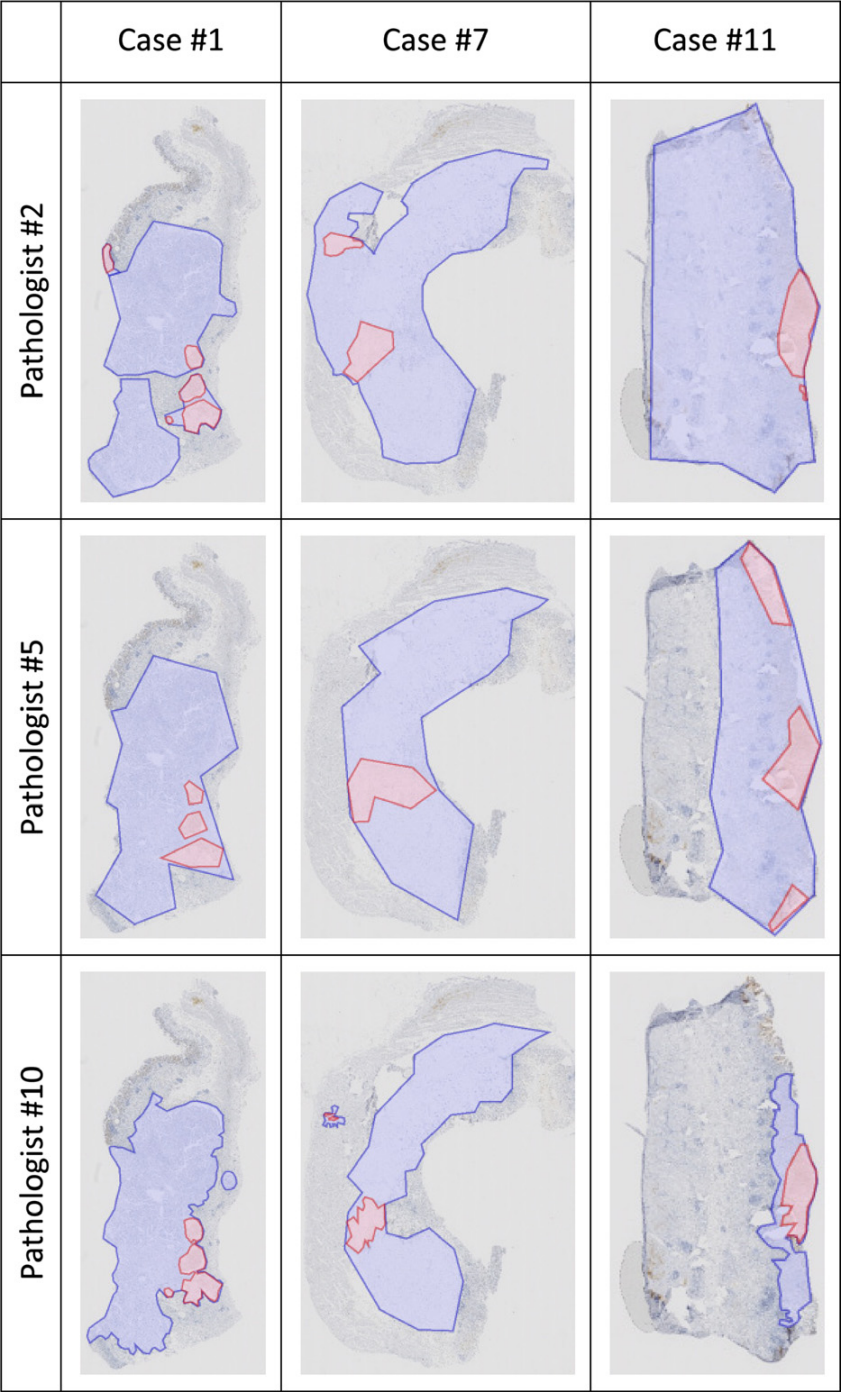
Examples of the outlines of tumor areas (pale blue) and positive tumor areas (pale red) that were manually drawn by three pathologists. They illustrate the variations in assessment of positive tumor areas, and also of total tumor area. Full data are shown in Table S2B.

**Figure 3:**
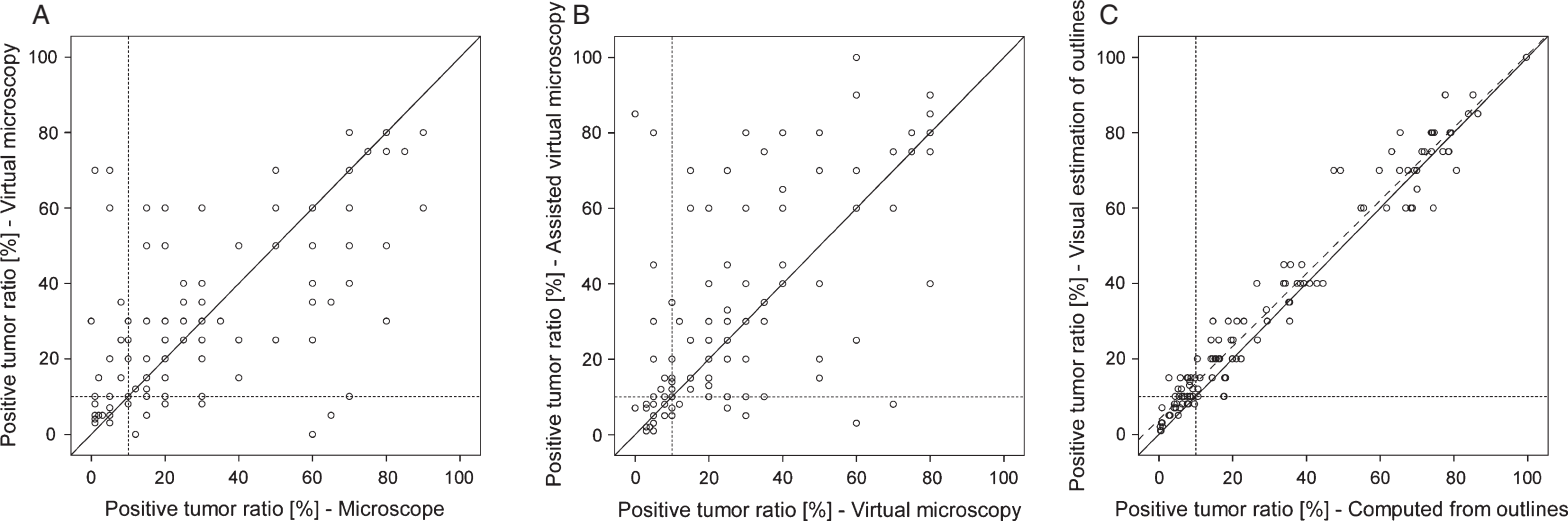
Comparison of positive tumor ratios between methods. Each data point represents two positive tumor ratios of the same case, rated by the same pathologist, using two methods. Solid diagonal lines mark complete agreement. Dotted lines denote 10% cutoff and isolate the discordant ratings in the upper left and lower right quadrant. (A) compares the microscopic method with virtual microscopy, and (B) compares virtual microscopy with assisted virtual microscopy. (C) How well the pathologists were able to estimate the positive tumor ratios of the outline sketches they had drawn, compared with their calculated ratios (Pearson's correlation coefficient was 0.974, *P* < 0.001.) Linear regression (dashed line) shows that positive tumor ratios were systematically overestimated.

The addition of manual area outlining prior to visual estimation of positive tumor ratio rendered several findings: First, there were differences in what the pathologists considered positively stained tumor, but also what they considered tumor tissue (Figs.2 and S2). Second, interobserver reproducibility (OCCC 0.672) was about the same as in microscopy, and slightly better than with plain virtual microscopy. Third, we found a nearly perfect correlation between the positive tumor ratio that was visually estimated from the outline sketches (Figs.2 and S2) and the ratios that were afterward calculated from the sketches (Fig.3C, Pearson's correlation coefficient 0.979, *P* < 0.001). This demonstrates that the pathologists are very well capable of estimating area ratios. However, we found 19 positive tumor ratios visually estimated on the outline sketches were above 10%, but turned out to be below 10% by calculation. Linear regression showed that the positive tumor ratio was visually overestimated compared with the calculated values. The overestimation is about 3.5% when the positive tumor ratio hovers around 10%. Thus, all 10 surgical pathologists (almost perfectly but systematically) overestimated the percentage of the positive tumor area.

An overview of the comparison of Her2/neu status of this study with the reference consensus Her2/neu status by the panel of three pathologists from the previous study 14 is given in Table3. Percentages of Her2/neu status ratings not concordant with the reference status ranged from 10% to 22.5% between the methods. We found discordances in six of 12 cases when rating was carried out using the microscope (Method 1). Application of virtual microscopy (Methods 2 and 3) showed a harmonizing effect by reducing the number of discordantly rated cases to 4. Interestingly, in case nos. 1 and 7 the computation of outlined areas from method 3 showed a positive tumor ratio below 10% in 19 of 20 outline drawings, indicating that probably the positive reference result was overestimated.

**Table 3:** Comparison of Her2/neu status results (10 individual pathologists) with consensus results from the previous study 14 (panel of three pathologists)

				Number of discordant ratings (10 individual pathologists)			
	Consensus results from the previous study 14 (panel of three pathologists)					Method 3: virtual microscopy with manual outlining	
Case no.	Her2/neu IRS	Her2/neu SISH	Her2/neu Status	Method 1: microscope	Method 2: virtual micropscopy	Visual estimation by pathologists	Computation of outlined areas
1	3+	Positive	Positive	1	2	3	10
2	3+	Positive	Positive	0	0	0	0
3	3+	Positive	Positive	0	0	0	0
4	2+	Negative	Negative	1	0	0	0
5	3+	Positive	Positive	0	0	0	0
6	3+	Positive	Positive	1	0	0	0
7	3+	Positive	Positive	6	6	8	10
8	0	Negative	Negative	0	0	0	0
9	3+	Positive	Positive	6	3	3	3
10	3+	Positive	Positive	0	0	0	0
11	3+	Positive	Positive	3	1	2	4
12	0	Negative	Negative	0	0	0	0
Total				18^1^ (15%)	12 (10%)	16 (13.3%)	27 (22.5%)

Except for one false-positive rating (case no. 4, Method 1), all discordant ratings were false-negative with respect to the consensus Her2/neu status. Total percentages of disagreements refer to the 120 ratings per method that were carried out by the 10 pathologists on the 12 slides.

1 17 false-negative and 1 false-positive rating.

### Agreements between methods

Agreements between methods were calculated using the Kappa test for staining intensities (Tables S2 and4), and OCCC for positive tumor ratios (Table4.) Additionally, we displayed the corresponding positive tumor ratio estimations from two methods, respectively, as scatter plots (Fig.3) and calculated Pearson's correlation coefficient.

**Table 4:** Intraobserver agreements, comparing two rating methods

	Agreement of positive tumor ratio			Agreement of staining intensity
Methods compared	OCCC	Pearson's correlation coefficient	Number of disagreements	Kappa
Microscopy versus virtual microscopy	0.647	0.651 (*P* < 0.001)	20	0.505 ± 0.068 (*P* < 0.001)
Plain virtual microscopy versus virtual microscopy with area outlining assistance	0.634	0.655 (*P* < 0.001)	18	0.509 ± 0.067 (*P* < 0.001)
Microscopy versus virtual microscopy with area outlining	0.665	0.680 (*P* < 0.001)	18	0.551 ± 0.066 (*P* < 0.001)

Agreements of positive tumor ratio were compared using OCCC, Pearson's correlation coefficient, and the number of discordant ratings with respect to the 10% cutoff. Agreements of staining intensities were calculated as kappa values.

Comparing Method 1 and Method 2 (microscope vs. virtual microscopy), intraobserver variation of positive tumor ratio showed a broad scattering (Fig.3A), accompanied by an OCCC of 0.647 and a Pearson's correlation coefficient of 0.651 (*P* < 0.001). Between Method 2 and Method 3 (plain virtual microscopy versus virtual microscopy with drawing of outlines) OCCC was 0.634 and Pearson's correlations coefficient was 0.655 (*P* < 0.001).

We counted the intraobserver disagreements whether positive tumor ratio was above or below 10% between methods (Fig.3). Between microscope and virtual microscopy we found 20 discordant estimations, 18 between plain and assisted virtual microscopy and 18 between microscopy and assisted virtual microscopy. Irrespective of the methodology, the intraobserver discordance always ranged between 15% and 17% and could not be improved by drawing outlines.

An additional hint that positive tumor areas are difficult to circumscribe could be found by comparing the interobserver agreements of the manually marked tumor areas and positive tumor areas: tumor areas could be more reliably delineated (OCCC was 0.807) than positive tumor areas (OCCC was 0.704).

Intraobserver comparisons of staining intensities are shown in Table S2. Between microscopy and virtual microscopy we found 84 matches (70%) and 36 mismatches (30%), as much as between plain and assisted microscopy. Between microscopy and assisted virtual microscopy there were 87 matches (72.5%) and 33 mismatches (27.5%). In all comparisons, the kappa value was between 0.505 and 0.551 (*P* < 0.001), indicating a moderate agreement.

These data show that intraobserver agreement declines when intensity of immunostaining is added to the scoring system. A thorough analysis of the individual drawings (see for instance Fig. S2, cases 6 and 12) illustrates that each pathologist draws unique outlines of what he/she considers to be the strongest identifiable immunostaining intensity, which then affects the overall IRS.

### Subgroup analysis

We divided the pathologists by their formal degree of experience into board-certified pathologists (*n* = 6) and residents (*n* = 4) to test whether experience has an influence on reproducibility. All calculations were repeated for both subgroups. Resulting data are shown in Tables S3 and S4. *P*-values of the tests remain significant. The measures of agreement of positive tumor ratio (i.e., mean of standard deviations, OCCC, and Pearson's correlation coefficient) vary between the subgroups, but we cannot identify a trend indicating an advantage for either subgroup. In contrast, inter- and intraobserver agreements of staining intensity show that board-certified pathologists can better reproduce staining intensities. Calculation of the interobserver agreements of the absolute tumor areas that were manually outlined in round 3 yielded an OCCC of 0.811 for the board-certified pathologists subgroup and 0.756 for the residents subgroup, respectively, showing a slight advantage for the board-certified pathologists. Agreement between visual estimation of area ratio of the manually drawn outlines and the corresponding computed values is nearly perfect (OCCC 0.966 and 0.983, respectively) for both subgroups. The number of specimens discordantly rated concerning the 10% cutoff could not be reduced using virtual microscopy in either group. The rate of false-negative ratings shows no clear advantage for either subgroup (Table S5).

## Discussion

For many years, only the anatomical location of the primary tumor, its histological phenotype and the tumor stage tailored chemotherapy. However, in clinical practice many patients with a seemingly identical tumor responded differently to the same therapy. Research on cancer biology provided ample explanations 21. Various genetic alterations and distinct molecular phenotypes were unraveled, which influence patient prognosis and response to chemotherapy. With the advent of targeted therapy, companion diagnostics is increasingly used to tailor patient treatment. Tumor-bearing tissue obtained prior to treatment is used to identify tumor-specific alterations (gene mutations, gene amplifications, protein expression-patterns), which predict therapeutic response. Testing the Her2/neu status has long been used in breast cancer. More recently, the To A-study provided strong evidence that Her2/neu overexpressing GCs also respond to treatment with trastuzumab. However, the assessment of Her2/neu overexpression is far more complicated in GC compared with breast cancer. First, it necessitated the development of a novel scoring system, which is different from the breast cancer scoring 22. GC cells more commonly harbor basolateral expression and rarely circumferential Her2/neu-staining 15,22. Second, expression of Her2/neu is heterogeneous bearing the risk of false-positive- and false-negative results due to sampling errors 14. In this study we examined the third pitfall, that is, the correct assessment of the cut-off value (i.e., 10%).

Our results demonstrate that pathologists are well capable estimating area ratios, independent of their experience, but with a slight systematic overestimation. It is the gradient in immunostaining intensity (“gray scale pattern”) that introduces errors by making it difficult to reliably delineate the borders between IRS values (e.g., 2+ vs. 3+). This difficulty is illustrated in Figure S4. Cases showing a sharply delineated “black-and-white” staining pattern can be rated easier and more reliably. Reproducibility and error rates highly depend on the individual staining pattern of each case. The situation is aggravated by the often heterogeneous distribution of positive tumor clones in GC, which introduces additional complexity to the staining pattern. This results in false-negative ratings, preventing an ISH analysis that could possibly render the patient eligible for Trastuzumab therapy. In our analysis, this false-negative rate was up to 19% (Table2). However, we had mainly selected cases known to be hovering around 10% for this study.

Knowledge of these sources of error is important because they may lead to the denial of Trastuzumab or, vice versa, to the prescription of medication without evidence of a treatment benefit. This will become even more important with the emergence of new targeted therapies using antibody-coupled drugs (e.g., Trastuzumab-Emtansine), which will need precise companion diagnostics with low false-negative and false-positive error rates. Future developments of immunohistochemistry-based companion diagnostics may wish to spend particular attention to heterogeneous biomarker expressions and the obstacles of a correct classification of percentage areas near a cut-off value.

How could the consequences of our observation be minimized? First, our trial to apply a computer-assisted method, namely manual outlining of the positive and total tumor areas within the virtual slide, did not help. A fully automated image analysis would presumably render higher precision and reproducibility, but not a more exact result as long as there are no well-defined on-slide references for calibration of Her2/neu staining intensities. Second, while we found that the reproducibility of positive tumor ratio estimation does not profit from experience, the rating of staining intensities does. Aside the existing recommendation, this gives further evidence that Her2/neu status should be evaluated by board-certified pathologists who are trained in the method. Third, cut-off limits should be established very carefully henceforth. There should be evidence for its necessity, its value and the viability of its measurement. Fourth, in cases having a positive tumor ratio hovering around 10% there should be a statement in the pathologic report that there is a chance of misinterpretation of the staining results. Anyway, in any case of doubt we would recommend to apply an ISH test.

It should be discussed whether to alter the rules for Her2/neu assessment in GC as it has recently be done for breast cancer 23, where cases with a strong staining and less than 10% positive tumor ratio are newly assigned to ISH testing to reduce the false-negative rate. To our knowledge, the 10% cutoff is not validated for GC, but rather adopted from the Her2/neu scoring rules for breast cancer.

Having available an H&E stained slide as a reference to help identify tumor areas might be helpful. In this study, the participants did not use H&E slides. Nevertheless, the overall agreement on what was tumor tissue (OCCC 0.807) was better than agreement on positively stained tumor areas (OCCC 0.704), so the larger source of errors certainly comes from the detection of stained areas and not from identifying tumor areas.

Another topic deserves attention: The Her2/neu testing recommendations require an additional ISH testing only in cases of equivocal, that is, IHC 2+, staining intensity, to distinguish positive from negative cases. IHC 1+ always counts as negative and IHC 3+ always as positive, without confirmation by ISH testing. A recent prospective study 24 has systematically investigated the relation between HER2 gene amplification and response to therapy in 66 cases of advanced GC. Using ROC analysis, an optimal HER2/CEP17 ratio threshold of 4.7 could be determined to predict response. It could also be predicted whether 12-month and 16-month survival was reached, respectively. This indicates the advantages of ISH testing, which is probably underestimated in the current recommendations, and might be considered for every case bearing any IHC staining greater than 0.

In conclusion, heterogeneous expression and distribution of predictive biomarkers in tumor tissue poses two major problems. First, nonrepresentative biopsy sampling leads to false-positive or -negative test results and may be overcome by analyzing greater numbers of biopsies or by combining the Her2/neu analysis of biopsy and surgical resection specimens 25. Second, the subjective assessment of an immunoreactivity IRS. Positive tumor ratios strongly depend on what each individual pathologist considers as maximum immunostaining intensity coupled with a systematical overestimation of tumor areas. Future developments of immunohistochemistry-based companion diagnostics may wish to spend particular attention to heterogeneous biomarker expressions and the obstacles of a correct classification of percentage areas near a cut-off value.

### Limitations

Our study could not assess the consequences for therapeutic outcome, because the patients of our cohort were only surgically resected and received no chemotherapy, neither adjuvant nor neoadjuvant.

## Conflict of Interest

None declared.

## Supporting Information

**Figure S1.** (A) Screenshot of our virtual microscopy program in outline-tracing mode. The pathologists manually marked tumor tissue (blue) and positive tumor tissue (red). (B) To test the ability of visually estimating area ratios, the pathologists were shown color-filled sketches of the regions they had outlined. These were only slightly tinted to avoid effects of color contrast or color perception. In a routine application one would not visually estimate the positive tumor ratios from drawn outlines because they could as well be computed; this only served to get an insight into the process of scoring.

**Figure S2.** Outlines manually drawn by the pathologists around areas of all tumor tissue (blue) and positive tumor tissue (red). Numbers below images denote the staining intensities from rating by microscope, by virtual microscopy, and by assisted virtual microscopy, respectively. Below are given the corresponding positive tumor ratios, plus the ratio that was calculated from the drawings. Continued on second page.

**Figure S3.** Areas of tumor tissue (blue) and positive tumor tissue (red) were calculated from the outline drawings, both given in square millimeters in the bar diagrams. The third row shows bar diagrams of the resulting, calculated positive tumor ratio. For direct comparison, in the bottom row the visually estimated positive tumor ratio from Figure 1 is repeated.

**Figure S4.** Illustration of 15% variability of intensity on a continuous gray scale at different levels. Each box spans 15% width of the gray scale. Viewing one box alone, the variation within is hardly noticeable.

**Table S1.** Clinical characteristics of the 12 cases of gastric cancer. UICC data (T, N, M, Stage) were recorded to seventh edition if only older data were available.

**Table S2.** Comparison of staining intensity ratings between methods. Numbers in the cross tables indicate the count of staining intensity combinations from two methods. Numbers on the diagonal (green cells) indicate agreement, and off-diagonal numbers indicate disagreement. (A) Agreement of staining intensity ratings between microscope and virtual microscopy. (B) Agreement of staining intensity ratings between virtual microscopy and assisted virtual microscopy. (C) Agreement of staining intensity ratings between microscope and assisted virtual microscopy

**Table S3.** Subgroup analysis of interobserver agreements between pathologists using one method of rating, respectively. Positive tumor ratios were compared using the mean of standard deviations, OCCC*), and number of discordant ratings with respect to the 10% cutoff. Staining intensities were compared using Fleiss’ kappa.

**Table S4.** Subgroup analysis of intraobserver agreements, comparing two rating methods, respectively. Agreements of positive tumor ratio were compared using OCCC, Pearson's correlation coefficient, and the number of discordant ratings with respect to the 10% cutoff. Agreements of staining intensities were calculated as kappa values.

**Table S5.** Subgroup analysis of the numbers of false-negative ratings and cases, respectively, due to underestimation of positive tumor area and/or staining intensity. Numbers in parentheses denote the false-negative percentage related to the total number of ratings that were done in each subgroup on the nine SISH-positive cases (72 ratings by six board-certified pathologists, and 48 ratings by four residents.)
