# Supplementary material for: Reproducibility of Her2/neu scoring in gastric cancer and assessment of the 10% cut-off rule
Source: Cancer Med. 2014 Dec 16;4(2):235–44. doi: 10.1002/cam4.365 (PMC4329007; doi:10.1002/cam4.365)

Supplemental Figure 1. **A**: Screenshot of our virtual microscopy program in outline-tracing mode. The pathologists manually marked tumor tissue (blue) and positive tumor tissue (red). **B**: To test the ability of visually estimating area ratios, the pathologists were shown color-filled sketches of the regions they had outlined. These were only slightly tinted to avoid effects of color contrast or color perception. In a routine application one would not visually estimate the positive tumor ratios from drawn outlines because they could as well be computed; this only served to get an insight in the process of scoring.

A **B**

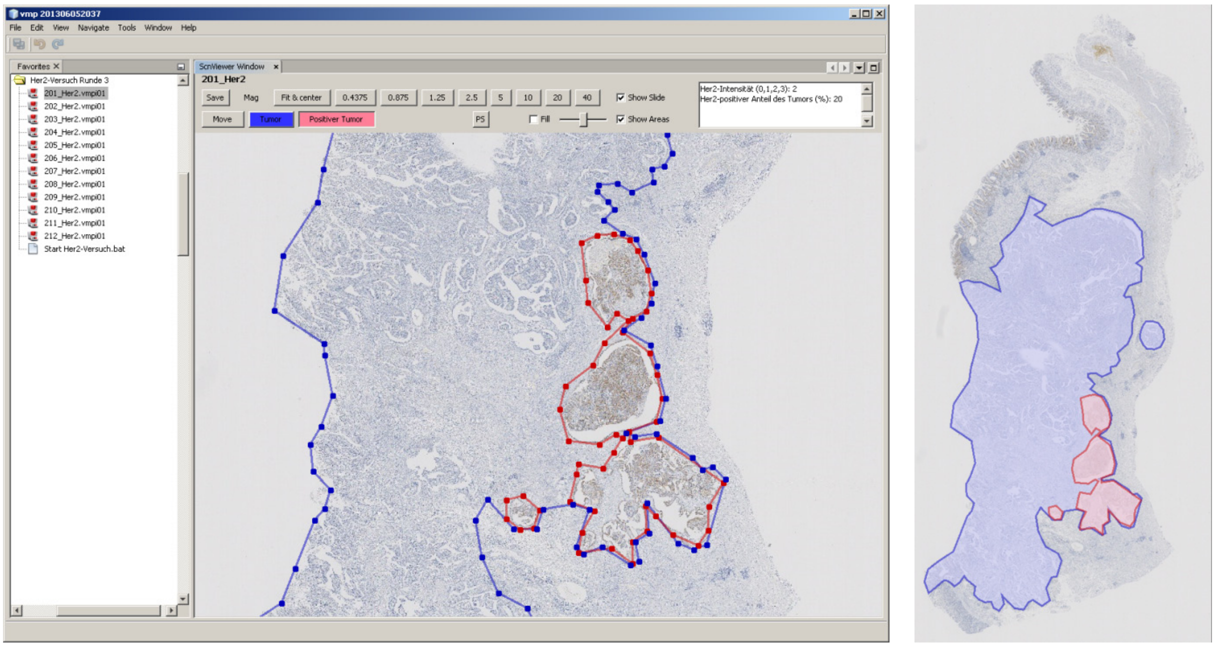

Supplement: Supplementary file 1 [file cam40004-0235-sd1.pdf]
