# Supplementary material for: Reproducibility of Her2/neu scoring in gastric cancer and assessment of the 10% cut-off rule
Source: Cancer Med. 2014 Dec 16;4(2):235–44. doi: 10.1002/cam4.365 (PMC4329007; doi:10.1002/cam4.365)
Supplement: Supplementary file 2 [file cam40004-0235-sd2.pdf]

**Supplemental Figure 2**. Outlines manually drawn by the pathologists around areas of all tumor tissue (blue) and positive tumor tissue (red). Numbers below images denote the staining intensities from rating by microscope, by virtual microscopy, and by assisted virtual microscopy, respectively. Below are given the corresponding positive tumor ratios, plus the ratio that was calculated from the drawings. Continued on second page.

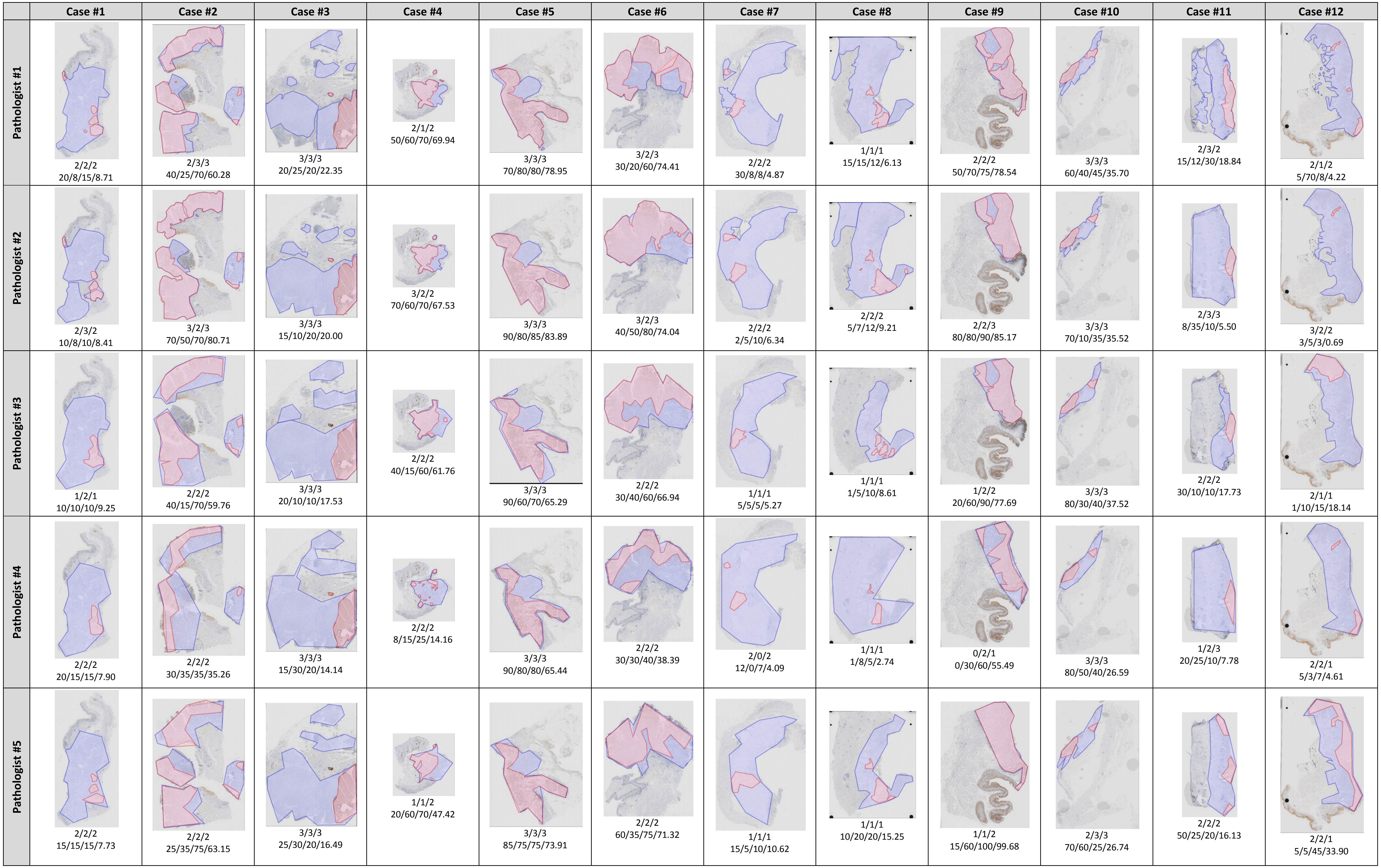

| | Case #1 | Case #2 | Case #3 | Case #4 | Case #5 | Case #6 | Case #7 | Case #8 | Case #9 | Case #10 | Case #11 | Case #12 |
|---|---|---|---|---|---|---|---|---|---|---|---|---|
| **Pathologist #1** | 2/2/2<br>20/8/15/8.71 | 2/3/3<br>40/25/70/60.28 | 3/3/3<br>20/25/20/22.35 | 2/1/2<br>50/60/70/69.94 | 3/3/3<br>70/80/80/78.95 | 3/2/3<br>30/20/60/74.41 | 2/2/2<br>30/8/8/4.87 | 1/1/1<br>15/15/12/6.13 | 2/2/2<br>50/70/75/78.54 | 3/3/3<br>60/40/45/35.70 | 2/3/2<br>15/12/30/18.84 | 2/1/2<br>5/70/8/4.22 |
| **Pathologist #2** | 2/3/2<br>10/8/10/8.41 | 3/2/3<br>70/50/70/80.71 | 3/3/3<br>15/10/20/20.00 | 3/2/2<br>70/60/70/67.53 | 3/3/3<br>90/80/85/83.89 | 3/2/3<br>40/50/80/74.04 | 2/2/2<br>2/5/10/6.34 | 2/2/2<br>5/7/12/9.21 | 2/2/3<br>80/80/90/85.17 | 3/3/3<br>70/10/35/35.52 | 2/3/3<br>8/35/10/5.50 | 3/2/2<br>3/5/3/0.69 |
| **Pathologist #3** | 1/2/1<br>10/10/10/9.25 | 2/2/2<br>40/15/70/59.76 | 3/3/3<br>20/10/10/17.53 | 2/2/2<br>40/15/60/61.76 | 3/3/3<br>90/60/70/65.29 | 2/2/2<br>30/40/60/66.94 | 1/1/1<br>5/5/5/5.27 | 1/1/1<br>1/5/10/8.61 | 1/2/2<br>20/60/90/77.69 | 3/3/3<br>80/30/40/37.52 | 2/2/2<br>30/10/10/17.73 | 2/1/1<br>1/10/15/18.14 |
| **Pathologist #4** | 2/2/2<br>20/15/15/7.90 | 2/2/2<br>30/35/35/35.26 | 3/3/3<br>15/30/20/14.14 | 2/2/2<br>8/15/25/14.16 | 3/3/3<br>90/80/80/65.44 | 2/2/2<br>30/30/40/38.39 | 2/0/2<br>12/0/7/4.09 | 1/1/1<br>1/8/5/2.74 | 0/2/1<br>0/30/60/55.49 | 3/3/3<br>80/50/40/26.59 | 1/2/3<br>20/25/10/7.78 | 2/2/1<br>5/3/7/4.61 |
| **Pathologist #5** | 2/2/2<br>15/15/15/7.73 | 2/2/2<br>25/35/75/63.15 | 3/3/3<br>25/30/20/16.49 | 1/1/2<br>20/60/70/47.42 | 3/3/3<br>85/75/75/73.91 | 2/2/2<br>60/35/75/71.32 | 1/1/1<br>15/5/10/10.62 | 1/1/1<br>10/20/20/15.25 | 1/1/2<br>15/60/100/99.68 | 2/3/3<br>70/60/25/26.74 | 2/2/2<br>50/25/20/16.13 | 2/2/1<br>5/5/45/33.90 |

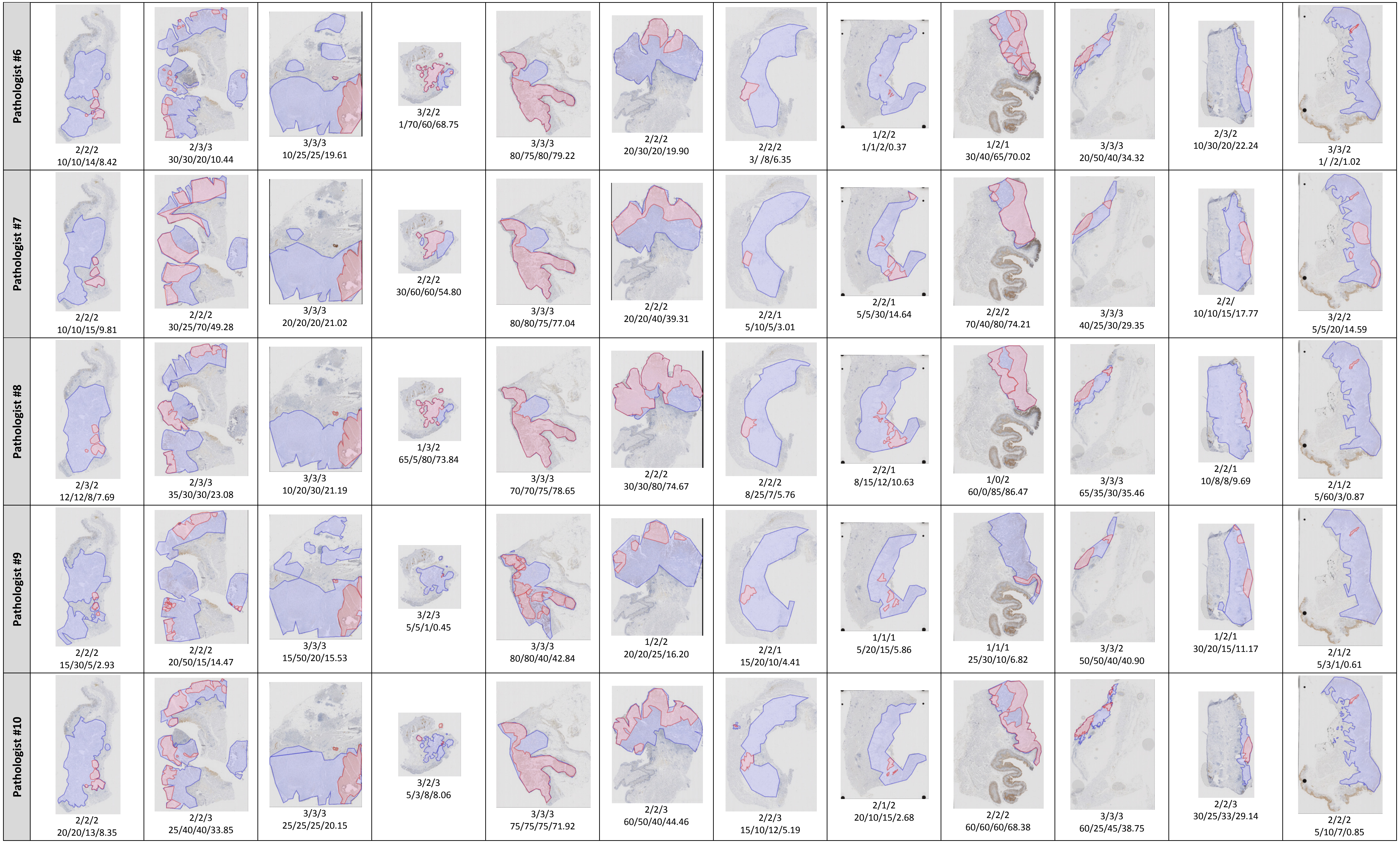

Pathologist #6
2/2/2 10/10/14/8.42
2/3/3 30/30/20/10.44
3/3/3 10/25/25/19.61
3/2/2 1/70/60/68.75
3/3/3 80/75/80/79.22
2/2/2 20/30/20/19.90
2/2/2 3/ /8/6.35
1/2/2 1/1/2/0.37
1/2/1 30/40/65/70.02
3/3/3 20/50/40/34.32
2/3/2 10/30/20/22.24
3/3/2 1/ /2/1.02
Pathologist #7
2/2/2 10/10/15/9.81
2/2/2 30/25/70/49.28
3/3/3 20/20/20/21.02
2/2/2 30/60/60/54.80
3/3/3 80/80/75/77.04
2/2/2 20/20/40/39.31
2/2/1 5/10/5/3.01
2/2/1 5/5/30/14.64
2/2/2 70/40/80/74.21
3/3/3 40/25/30/29.35
2/2/ 10/10/15/17.77
3/2/2 5/5/20/14.59
Pathologist #8
2/3/2 12/12/8/7.69
2/3/3 35/30/30/23.08
3/3/3 10/20/30/21.19
1/3/2 65/5/80/73.84
3/3/3 70/70/75/78.65
2/2/2 30/30/80/74.67
2/2/2 8/25/7/5.76
2/2/1 8/15/12/10.63
1/0/2 60/0/85/86.47
3/3/3 65/35/30/35.46
2/2/1 10/8/8/9.69
2/1/2 5/60/3/0.87
Pathologist #9
2/2/2 15/30/5/2.93
2/2/2 20/50/15/14.47
3/3/3 15/50/20/15.53
3/2/3 5/5/1/0.45
3/3/3 80/80/40/42.84
1/2/2 20/20/25/16.20
2/2/1 15/20/10/4.41
1/1/1 5/20/15/5.86
1/1/1 25/30/10/6.82
3/3/2 50/50/40/40.90
1/2/1 30/20/15/11.17
2/1/2 5/3/1/0.61
Pathologist #10
2/2/2 20/20/13/8.35
2/2/3 25/40/40/33.85
3/3/3 25/25/25/20.15
3/2/3 5/3/8/8.06
3/3/3 75/75/75/71.92
2/2/3 60/50/40/44.46
2/2/3 15/10/12/5.19
2/1/2 20/10/15/2.68
2/2/2 60/60/60/68.38
3/3/3 60/25/45/38.75
2/2/3 30/25/33/29.14
2/2/2 5/10/7/0.85
