# Supplementary material for: Reproducibility of Her2/neu scoring in gastric cancer and assessment of the 10% cut-off rule
Source: Cancer Med. 2014 Dec 16;4(2):235–44. doi: 10.1002/cam4.365 (PMC4329007; doi:10.1002/cam4.365)

Supplemental Figure 3. Areas of tumor tissue (blue) and positive tumor tissue (red) were calculated from the outline drawings, both given in square millimeters in the bar diagrams. The third row shows bar diagrams of the resulting, calculated positive tumor ratio. For direct comparison, in the bottom row the visually estimated positive tumor ratio from Figure 1 is repeated.

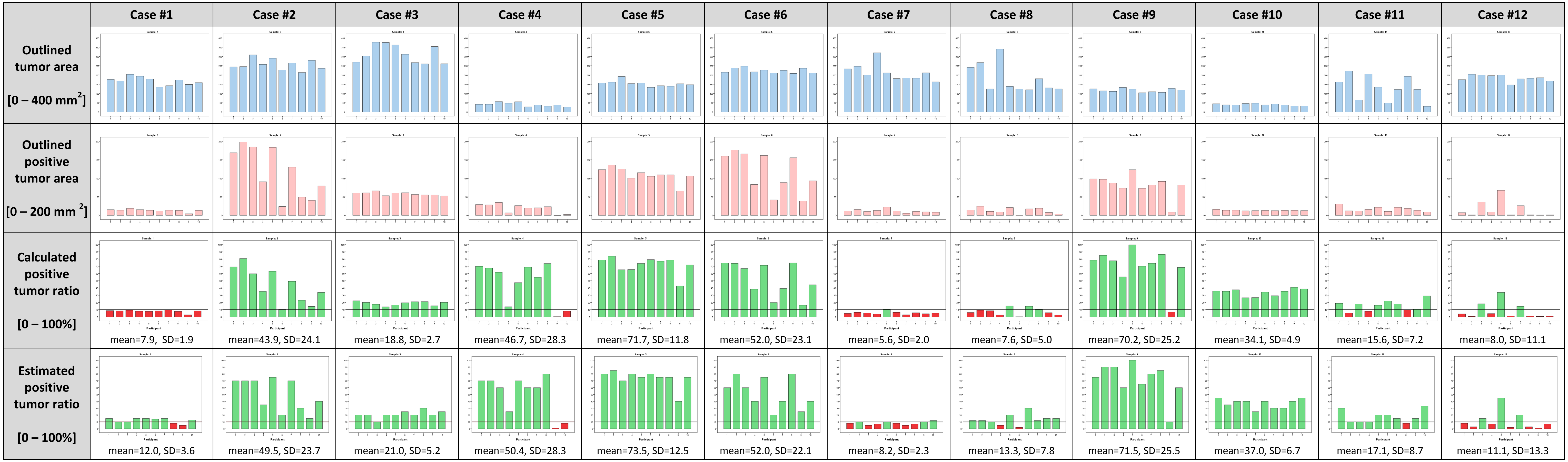

Supplement: Supplementary file 3 [file cam40004-0235-sd3.pdf]
