# Supplementary material for: Reproducibility of Her2/neu scoring in gastric cancer and assessment of the 10% cut-off rule
Source: Cancer Med. 2014 Dec 16;4(2):235–44. doi: 10.1002/cam4.365 (PMC4329007; doi:10.1002/cam4.365)

Supplemental Figure 4. Illustration of 15% variability of intensity on a continuous gray scale at different levels. Each box spans 15% width of the gray scale. Viewing one box alone, the variation within is hardly noticeable.

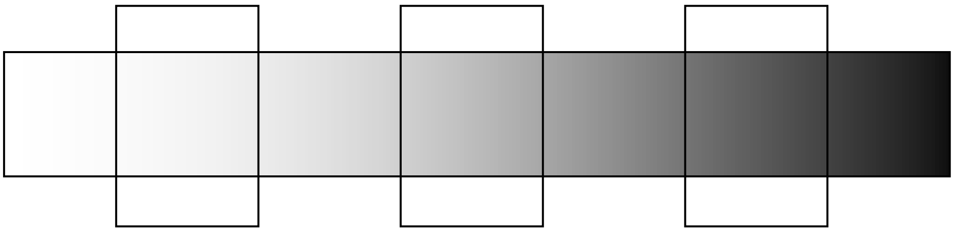

Supplement: Supplementary file 4 [file cam40004-0235-sd4.pdf]
