# Supplementary material for: Reproducibility of Her2/neu scoring in gastric cancer and assessment of the 10% cut-off rule
Source: Cancer Med. 2014 Dec 16;4(2):235–44. doi: 10.1002/cam4.365 (PMC4329007; doi:10.1002/cam4.365)
Supplement: Supplementary file 5 [file cam40004-0235-sd5.pdf]

Supplemental Table 1. Clinical characteristics of the 12 cases of gastric cancer. UICC data (T, N, M, Stage) were recoded to 7th edition if only older data was available.

Case No.	Gender	Age at diagnosis [years]	Localization	Laurén type	T	N	M	UICC Stage	Kiel Stage	Her2/neu IRS *)	Her2/neu SISH *)	Her2/neu Status *)
1	Male	77	Distal stomach	Intestinal	T2	N1	M0	IIA	IIIA	3+	positive	positive
2	Female	75	Proximal stomach	Intestinal	T2	N0	M0	IB	II	3+	positive	positive
3	Male	42	Proximal stomach	Intestinal	T3	N3	M1	IV	IV	3+	positive	positive
4	Female	85	Distal stomach	Intestinal	T1b	N0	M0	IA	I	2+	negative	negative
5	Male	69	Distal stomach	Intestinal	T3	N3a	M0	IIIB	IIIB	3+	positive	positive
6	Female	78	Proximal stomach	Intestinal	T4a	N3	M1	IV	IV	3+	positive	positive
7	Male	66	Distal stomach	Intestinal	T2	N0	M0	IB	II	3+	positive	positive
8	Male	78	Distal stomach	Mixed	T4a	N2	M0	IIIB	IIIB	0	negative	negative
9	Female	68	Proximal stomach	Intestinal	T2	N0	M0	IB	II	3+	positive	positive
10	Male	62	Proximal stomach	Intestinal	T1b	N2	M0	IIA	IIIB	3+	positive	positive
11	Female	68	Distal stomach	Diffuse	T4a	N3a	M0	IIIC	IV	3+	positive	positive
12	Male	80	Distal stomach	Intestinal	T3	N1	M0	IIB	IIIA	0	negative	negative

*) Consensus Her2/neu results retrieved from the previous study[14]
