# Supplementary material for: Reproducibility of Her2/neu scoring in gastric cancer and assessment of the 10% cut-off rule
Source: Cancer Med. 2014 Dec 16;4(2):235–44. doi: 10.1002/cam4.365 (PMC4329007; doi:10.1002/cam4.365)
Supplement: Supplementary file 6 [file cam40004-0235-sd6.pdf]

Supplemental Table 2. Comparison of staining intensity ratings between methods. Numbers in the cross tables indicate the count of staining intensity combinations from two methods. Numbers on the diagonal (green cells) indicate agreement, and off-diagonal numbers indicate disagreement.

A) Agreement of staining intensity ratings between microscope and virtual microscopy

		SIS round 2 (screen)				
		0	1	2	3	Total
SIS round 1 (microscope)	0	0	0	1	0	1
	1	1	10	7	1	19
	2	1	6	45	10	62
	3	0	0	9	29	38
	Total	2	16	62	40	120

84 matches (70%) and 36 mismatches (30%)
Kappa = 0.505±0.068 ($p<0.001$)

B) Agreement of staining intensity ratings between virtual microscopy and assisted virtual microscopy

		SIS round 3 (screen, repetition)				
		0	1	2	3	Total
SIS round 2 (screen)	0	0	0	2	0	2
	1	0	9	7	0	16
	2	0	11	42	9	62
	3	0	0	7	33	40
	Total	0	20	58	42	120

84 matches (70%) and 36 mismatches (30%)
Kappa = 0.509±0.067 ($p<0.001$)

C) Agreement of staining intensity ratings between microscope) and assisted virtual microscopy

		SIS round 3 (screen, repetition)				
		0	1	2	3	Total
SIS round 1 (microscope)	0	0	1	0	0	1
	1	0	11	8	0	19
	2	0	8	44	10	62
	3	0	0	6	32	38
	Total	0	20	58	42	120

87 matches (72.5%) and 33 mismatches (27.5%)
Kappa = 0.551±0.066 ($p<0.001$)
