# Supplementary material for: Reproducibility of Her2/neu scoring in gastric cancer and assessment of the 10% cut-off rule
Source: Cancer Med. 2014 Dec 16;4(2):235–44. doi: 10.1002/cam4.365 (PMC4329007; doi:10.1002/cam4.365)
Supplement: Supplementary file 7 [file cam40004-0235-sd7.pdf]

Supplemental Table 3. Subgroup analysis of inter-observer agreements between pathologists using one method of rating, respectively. Positive tumor ratios were compared using the mean of standard deviations, OCCC*), and number of discordant ratings with respect to the 10% cutoff. Staining intensities were compared using Fleiss' kappa.

	Board-certified pathologists (n=6)				Residents (n=4)			
	Agreement of positive tumor ratio			Agreement of staining intensity	Agreement of positive tumor ratio			Agreement of staining intensity
**Method**	**OCCC*)**	**Mean of standard deviations [%] (range)**	**Number of specimens with 10% cut-off disagreements**	**Fleiss' kappa**	**OCCC*)**	**Mean of standard deviations [%] (range)**	**Number of specimens with 10% cut-off disagreements**	**Fleiss' kappa**
Microscope	0.688	11.4	4	0.47 (p<0.001)	0.723	12.2	4	0.36 (p<0.001)
Virtual microscopy	0.632	11.8	5	0.45 (p<0.001)	0.466	14.2	7	0.37 (p<0.001)
Virtual microscopy with outlining assistance and visual estimation of positive tumor ratio	0.574	14.3	5	0.51 (p<0.001)	0.774	11.0	5	0.24 (p<0.001)
Virtual microscopy with outlining assistance and calculation of positive tumor ratio	0.600	13.5	6		0.799	9.5	4	

*) Overall concordance correlation coefficient
