# Supplementary material for: Reproducibility of Her2/neu scoring in gastric cancer and assessment of the 10% cut-off rule
Source: Cancer Med. 2014 Dec 16;4(2):235–44. doi: 10.1002/cam4.365 (PMC4329007; doi:10.1002/cam4.365)
Supplement: Supplementary file 8 [file cam40004-0235-sd8.pdf]

Supplemental Table 4. Subgroup analysis of intra-observer agreements, comparing two rating methods, respectively. Agreements of positive tumor ratio were compared using OCCC, Pearson's correlation coefficient, and the number of discordant ratings with respect to the 10% cutoff. Agreements of staining intensities were calculated as kappa values.

	Board-certified pathologists (n=6)				Residents (n=4)			
	Agreement of positive tumor ratio			Agreement of staining intensity	Agreement of positive tumor ratio			Agreement of staining intensity
**Methods compared**	**OCCC**	**Pearson's correlation coefficient**	**Number of disagreements**	**Kappa**	**OCCC**	**Pearson's correlation coefficient**	**Number of disagreements**	**Kappa**
Microscopy versus virtual microscopy	0.687	0.698 (p<0.001)	10	0.548±0.086 (p<0.001)	0.605	0.630 (p<0.001)	10	0.435±0.111 (p<0.001)
Plain virtual microscopy versus virtual microscopy with area outlining assistance	0.674	0.682 (p<0.001)	8	0.568±0.084 (p<0.001)	0.587	0.639 (p<0.001)	10	0.417±0.111 (p<0.001)
Microscopy versus virtual microscopy with area outlining	0.582	0.603 (p<0.001)	10	0.592±0.083 (p<0.001)	0.760	0.766 (p<0.001)	8	0.484±0.108 (p<0.001)
