# Supplementary material for: Reproducibility of Her2/neu scoring in gastric cancer and assessment of the 10% cut-off rule
Source: Cancer Med. 2014 Dec 16;4(2):235–44. doi: 10.1002/cam4.365 (PMC4329007; doi:10.1002/cam4.365)
Supplement: Supplementary file 9 [file cam40004-0235-sd9.pdf]

Supplemental Table 5. Subgroup analysis of the numbers of false-negative ratings and cases, respectively, due to underestimation of positive tumor area and/or staining intensity. Numbers in parentheses denote the false-negative percentage related to the total number of ratings that were done in each subgroup on the 9 SISH-positive cases (72 ratings by 6 board-certified pathologists, and 48 ratings by 4 residents.)

	**Board-certified pathologists (n=6)**			**Residents (n=4)**		
	Number of false-negative ratings / number of cases affected			Number of false-negative ratings / number of cases affected		
	Microscope	Virtual microscopy	Assisted virtual microscopy	Microscope	Virtual microscopy	Assisted virtual microscopy
False-negative because positive tumor ratio was underestimated (<10%), while IRS was 2+ or 3+ and SISH was positive	2 (3%) / 1	3 (4%) / 2	4 (6%) / 2	4 (8%) / 2	3 (6%) / 3	2 (4%) / 2
False-negative because IRS was underestimated (0 or 1+), while positive tumor ratio ≥10% and SISH positive	8 (11%) / 5	2 (3%) / 1	6 (8%) / 3	3 (6%) / 2	0 / 0	1 (2%) / 1
Total false-negative rate because positive tumor ratio or IRS or both were underestimated, while SISH was positive	10 (14%) / 4	7 (10%) / 3	11 (15%) / 4	7 (15%) / 4	5 (10%) / 4	5 (10%) / 3
